# Comprehensive deep learning-assisted multi-condition analysis of knee MRI studies improves resident radiologist performance

**DOI:** 10.1007/s00330-025-12052-8

**Published:** 2025-10-17

**Authors:** Roman Vuskov, Alexander Hermans, Martin Pixberg, Jonas Müller-Hübenthal, Andreas Brauksiepe, Eric Corban, Malin Cubukcu, Julia Nowak, Aleksandar Kargaliev, Marc von der Stück, Robert Siepmann, Christiane Kuhl, Daniel Truhn, Sven Nebelung

**Affiliations:** 1 Lab for Artificial Intelligence in Medicine, Department of Diagnostic and Interventional Radiology, University Hospital RWTH Aachen, Aachen, Germany; 2 Department of Diagnostic and Interventional Radiology, University Hospital RWTH Aachen, Aachen, Germany; 3 Visual Computing Institute (Computer Vision), RWTH Aachen University, Aachen, Germany; 4 Radiologic Practice Cologne Triangle, Cologne, Germany

**Keywords:** Knee, Magnetic resonance imaging, Deep learning

## Abstract

**Objectives:**

Developing a deep-learning model for automated multi-tissue, multi-condition knee MRI analysis and assessing its clinical potential.

**Material and methods:**

This retrospective dual-center study included 3121 MRI studies from 3018 adults, who underwent routine knee MRI examinations at a radiologic practice (2012–2019). Twenty-three conditions across cartilage, menisci, bone marrow, ligaments, and other soft tissues were manually labeled. A 3D slice transformer network was trained for binary classification and evaluated in terms of the area under the receiver operating characteristic curve (AUC), sensitivity, and specificity using a five-fold cross-validation and an external test set of 448 MRI studies (429 adults) from a university hospital (2022–2023). To assess differences in diagnostic performance, two inexperienced and two experienced radiology residents read 50 external test studies with and without model assistance. Paired *t*-tests were used for statistical analysis.

**Results:**

Averaged over cross-validation tests, the model’s AUC was at least 0.85 for 8 conditions and at least 0.75 for 18 conditions. Generalization on the external test set was robust, with a mean absolute AUC difference of 0.05 ± 0.03 per condition. Model assistance improved accuracy and sensitivity for inexperienced residents, increased inter-reader agreement for both groups, and increased sensitivity and shortened reading times by 10% (*p* = 0.045) for experienced residents. Specificity decreased slightly when conditions with low model performance (AUC < 0.75) were included.

**Conclusion:**

Our deep-learning model performed well across diverse knee conditions and effectively assisted radiology residents. Future work should focus on more fine-grained predictions for subtle or rare conditions to enable comprehensive joint assessment in clinical practice.

**Key Points:**

***Question**** Increasing MRI utilization adds pressure on radiologists, necessitating comprehensive AI models for image analysis to manage this growing demand efficiently*.

***Findings**** Our AI model enhanced diagnostic performance and efficiency of resident radiologists when reading knee MRI studies, demonstrating robust results across diverse conditions and two datasets*.

***Clinical relevance**** Model assistance increases the sensitivity of radiologists, helping to identify pathologies that were overlooked without AI assistance. Reduced reading times suggest potential alleviation of radiologists’ workload*.

**Graphical Abstract:**

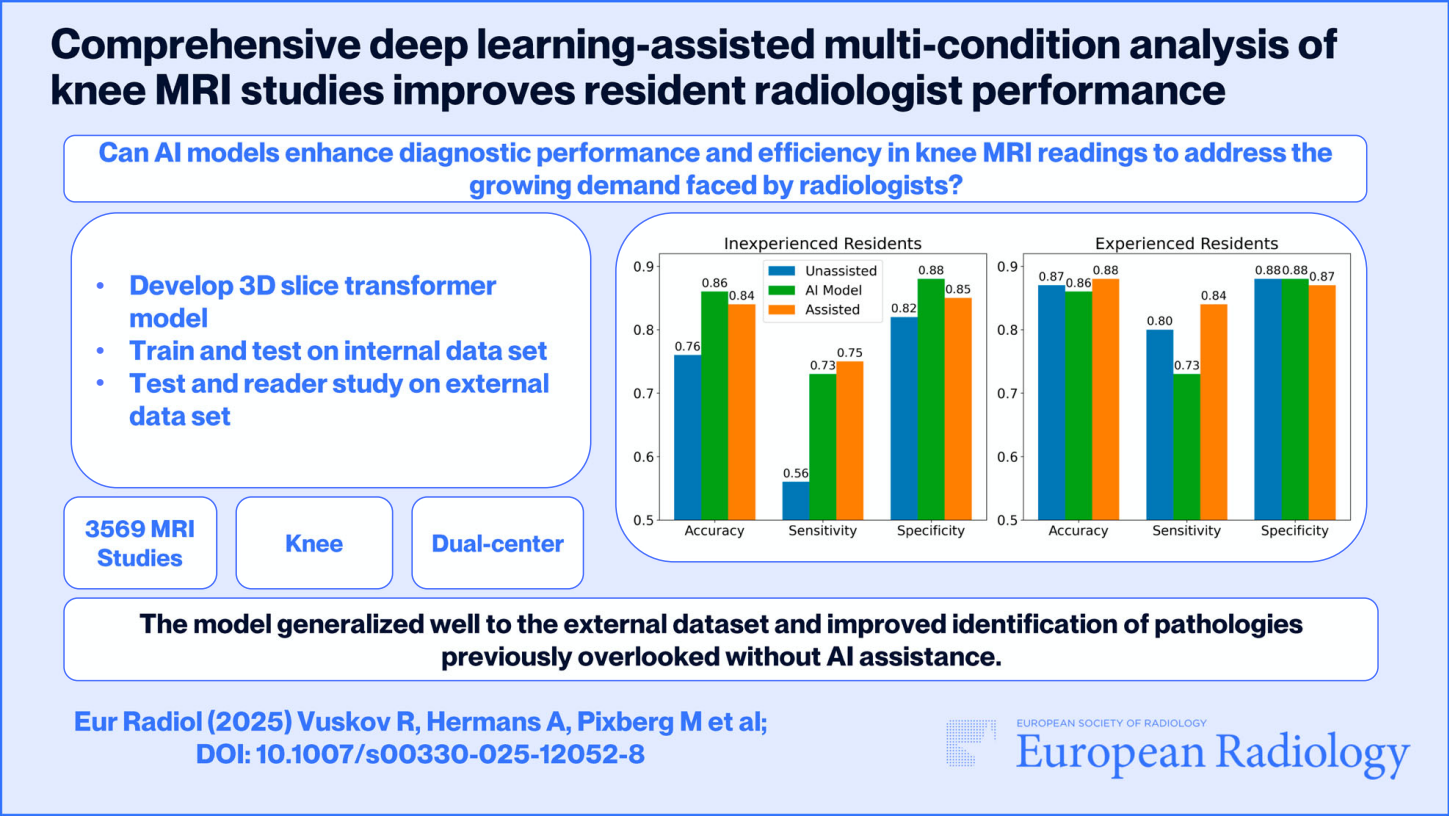

## Introduction

MRI utilization rates steadily increase globally [1], with average utilization exceeding 100 MRI studies per year per 1000 inhabitants in many Western countries [2]. Concurrent with this surge in MRI utilization is the increasing complexity of each MRI study, not least due to the higher numbers of images, which translates into substantial increases in image content per MRI study [3, 4]. While MRI studies undoubtedly improve patient care by facilitating more accurate and earlier diagnoses, increasing study complexity and volume increases radiologists’ workload and challenges in upholding diagnostic quality. Higher workloads are only partially mitigated by increased staffing levels and may predispose to deteriorating diagnostic performance, increased error rates, extended working hours, and a higher prevalence of burnout and fatigue [5]. This problem is further exacerbated by recent developments that substantially increase patient throughput in radiologic practices. In particular, the increasing implementation of AI-accelerated image acquisitions renders previously inconceivable 5-min protocols—for the knee and beyond—a clinical reality [6, 7]. Combined with the increasing clinical demand for knee MRI, considered the “ultimate imaging modality” [4, 8], AI-based image analysis has yet to keep up [9]. Most previous studies have focused on single tissues or single conditions [10–13], while preliminary multi-condition approaches used research sequences and study cohorts unreflective of clinical reality [11].

Consequently, there is an unmet clinical need for AI models that provide comprehensive diagnostic assistance for knee MRI, aiding radiologists in managing the increasing amount of image content to read and interpret. Our objective was to develop an AI model for assisted readings of knee MRI studies and to evaluate its potential value for resident radiologists. We hypothesized that such a model would (i) demonstrate sufficient diagnostic performance in identifying common knee conditions, (ii) generalize well to external data, and (iii) assist residents in reading knee MRI studies more accurately and efficiently.

## Material and methods

### Study design

This retrospective study was conducted following local data protection regulations. After approval by the local Ethical Committee (Medical Faculty, RWTH Aachen University (Germany): EK 22-319), the requirement for individual informed consent was waived.

### Dataset characteristics

**Fig. 1:**
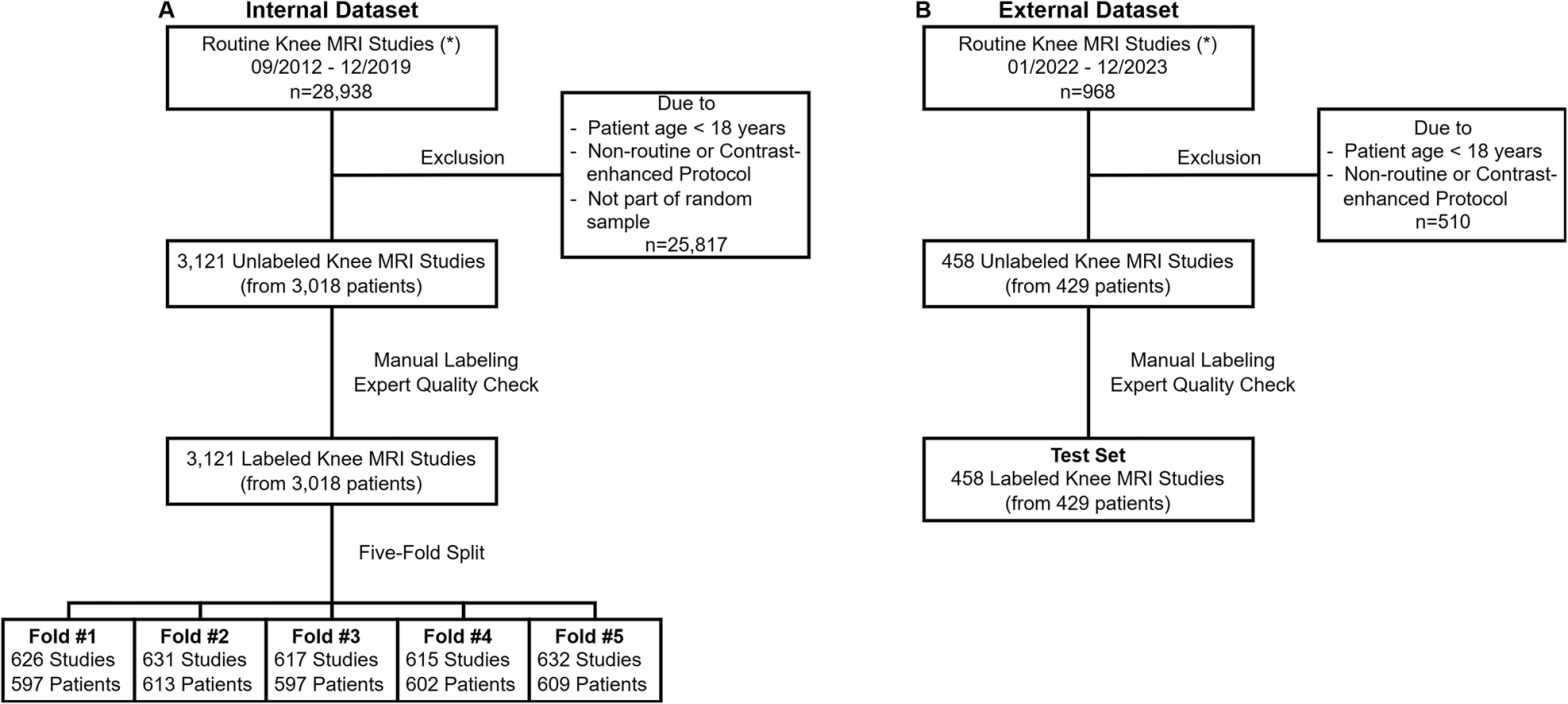
Workflow for internal (**A**) and external (**B**) datasets. After random sampling and applying the exclusion criteria, routine knee MRI studies were manually labeled regarding the presence or absence of 23 relevant conditions. Model performance was assessed with five-fold cross-validation using the internal dataset (**A**) and an external held-out test set (**B**). (*) Routine knee MRI studies were non-enhanced and consisted of axial, coronal, and sagittal proton density-weighted fat-saturated sequences and a sagittal T1-weighted sequence

This study used two datasets of non-contrast-enhanced knee MRI studies acquired as part of the clinical routine from two radiologic institutions (Fig. 1). The first dataset came from a private radiologic practice (Praxis im Köln Triangle, Cologne, Germany) and consisted of 3121 MRI studies from 3018 patients acquired between 09/2012 and 12/2019. This dataset was used for model training, validation, and (internal) testing (*internal dataset*). The second dataset came from a university hospital (Aachen, Germany), consisting of 458 MRI studies from 429 patients acquired between 01/2022 and 12/2023. This dataset was used for external testing (*external dataset*).

### Internal dataset

For the specified period, MRI studies consisting of axial, coronal, and sagittal proton density-weighted fat-saturated and sagittal T1-weighted 2D turbo-spin echo sequences of adult patients were randomly sampled from the local PACS (Centricity, GE HealthCare). No specific exclusion criteria were applied to reflect the clinical routine. Two MRI scanners were used for image acquisition: a 1.5-T scanner (Intera, Philips Medical Systems) and a 3-T scanner (Magnetom Skyra, Siemens Healthineers). Supplementary Table 1 details the acquisition parameters.

Three board-certified general radiologists with a primary focus on MSK radiology and at least 10 years of clinical experience read the MRI studies as part of their clinical routine. Reporting was based on the essential and supporting diagnostic criteria in Supplementary Table 2 and provided the basis for subsequent patient care. Four pre-graduate medical students manually extracted labels using the final radiologic reports and referred to the MRI studies when needed. Following meticulous training—supplemented by online Q&A documentation and regular feedback sessions—the students used a structured template consisting of 23 conditions to assign binary labels for each MRI study, indicating the presence or absence of each condition (Table 1). These conditions included (i) tears and non-tear pathologies of the cruciate and collateral ligaments, (ii) meniscal tears, extrusion, and degenerative meniscopathy, (iii) cartilage pathologies, and (iv) other conditions such as bone marrow edema, joint effusion (including Baker’s cysts), prepatellar bursitis, and off-position patellae. Two additional board-certified general radiologists who had not been involved in the original reporting reviewed all labels to ensure accuracy and consistency.

**Table 1:** Dataset characteristics and condition counts

Characteristic	Internal data set	External data set
Patient demographics		
Unique patients (*n*)	3018	429
Age (years)	45 ± 16	43 ± 16
Female	1524 (50.5)	192 (44.8)
Male	1494 (49.5)	237 (55.3)
MRI studies (*n*)	3121	458
St. p. ACL Reconstruction	159 (5.1)	36 (7.9)
MCL tear (partial/complete)	83 (2.7)	12 (2.6)
Baker’s cyst	849 (27.2)	71 (15.5)
Effusion	973 (31.2)	190 (41.5)
Lateralization	160 (5.1)	19 (4.1)
ACL tear (partial/complete)	162 (5.2)	52 (11.4)
Medial meniscus extrusion	1009 (32.3)	59 (12.9)
Medial meniscus tear	892 (28.6)	133 (29.0)
Prepatellar bursitis	83 (2.7)	2 (0.4)
Lateral meniscus extrusion	275 (8.8)	28 (6.1)
Tibial cartilage pathology	708 (22.7)	114 (24.9)
Retropatellar cartilage pathology	1183 (37.9)	204 (44.5)
Lateral meniscus tear	264 (8.5)	34 (7.4)
Femoral cartilage pathology	1317 (42.2)	230 (50.2)
Bone marrow edema	1267 (40.6)	252 (55.0)
Patella alta	133 (4.3)	8 (1.7)
ACL pathology (non-tear)	391 (12.5)	34 (7.4)
Lateral meniscus degeneration	403 (12.9)	66 (14.4)
MCL pathology (non-tear)	237 (7.6)	11 (2.4)
LCL pathology (non-tear)	379 (12.1)	3 (0.7)
LCL tear (partial/complete)	15 (0.5)	11 (2.4)
Medial meniscus degeneration	546 (17.5)	147 (32.1)
PCL pathology (tear & non-tear)	63 (2.0)	19 (4.1)

Except for age, which is indicated as mean ± standard deviation, values are numbers of positive counts (percentages)

*St. p*. Status post,* ACL* anterior cruciate ligament, *MCL* medial collateral ligament, *LCL* lateral collateral ligament, *PCL* posterior cruciate ligament

The dataset was split at the patient level into five folds of similar size and distribution using stratified random sampling (Supplementary Table 3).

### External dataset

All consecutive adult patients with a routine non-contrast enhanced knee protocol as above were included, and no additional exclusion criteria were applied. Studies from four MRI scanners (from Philips) were available: two 1.5-T scanners (Ingenia, Ingenia Ambition X) and two 3-T scanners (Ingenia Elition X, Achieva) (Supplementary Table 1). 14 board-certified attending radiologists, all of whom had advanced expertise in clinical MRI but varying levels of clinical experience, reported on the MRI studies.

Labels were manually extracted, and quality was checked as detailed above.

### Data preprocessing

Histogram normalization [14] was applied to each image sequence separately. The normalization parameters were calculated using the internal dataset and then applied to the internal and external datasets. The resulting intensities were clipped at their respective 99.9^th^ percentiles and linearly rescaled to [0,1]. Then, normalization using the ImageNet mean and variance was applied [15]. Each image stack was trilinearly resized to 256 × 256 pixels in-plane and 32 slices through-plane to improve training efficiency and model convergence. The number of images was fixed at 32 because 99% of the datasets used for training contained fewer than 33 images. During training, each image stack was subjected to augmentations (Supplementary Table 4).

### Model development

**Fig. 2:**
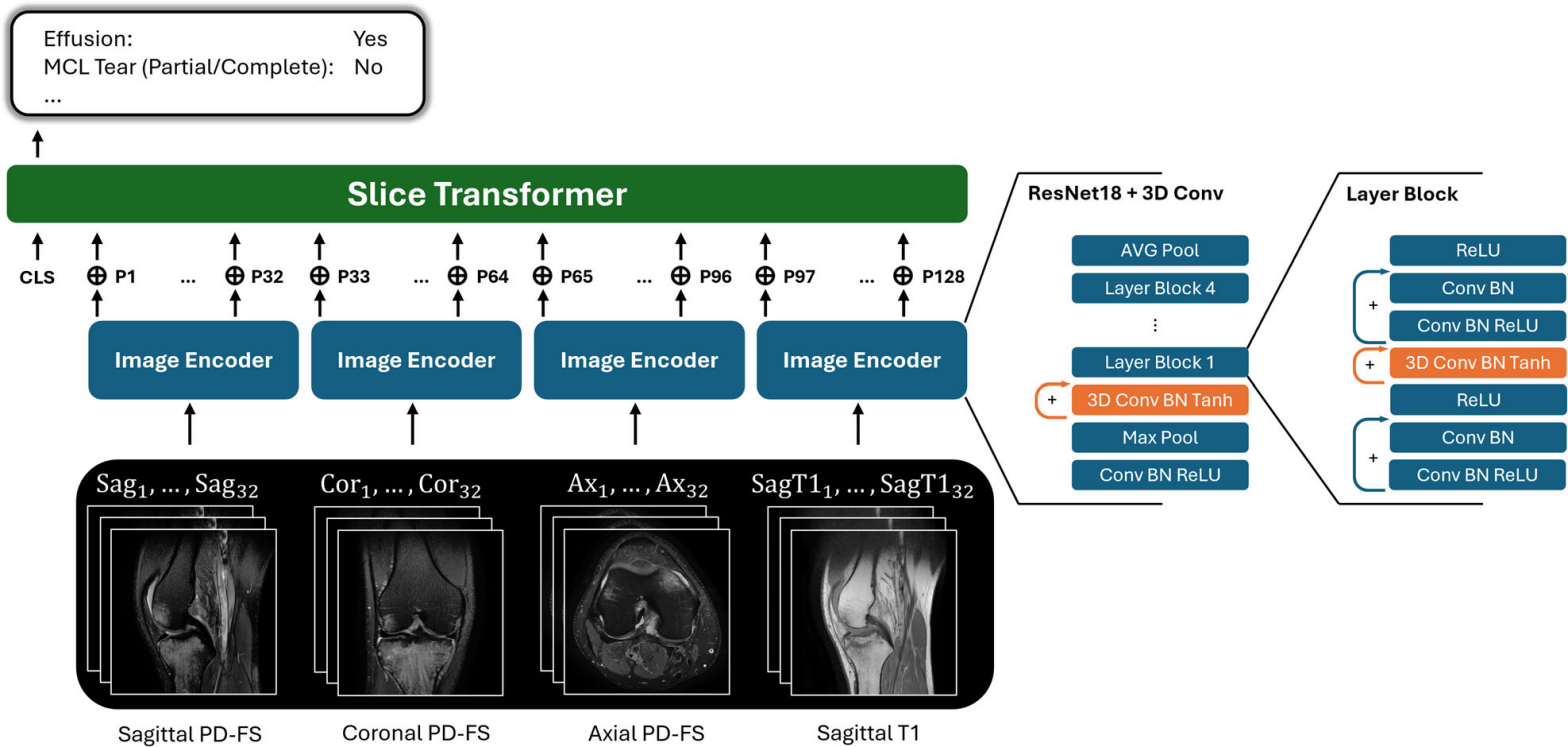
Deep-learning model architecture. The deep-learning model processes clinical routine knee MRI studies, i.e., sagittal, coronal, axial proton density-weighted (PD) 2D turbo spin-echo sequences with fat saturation (FS), and a sagittal T1-weighted sequence. Each sequence is resized to 256×256 pixels and 32 slices and processed using a modified ResNet18 backbone (schematically detailed on the right). The backbone integrates residual 3D convolutions (orange) with standard ResNet18 blocks (blue) and encodes each slice into a single token while preserving contextual information from adjacent slices. Positional embeddings are added to the token sequence, and a class token is prepended before being fed into an encoder-only transformer for classification. The output predicts the presence or absence of 23 conditions of the knee joint. Arrows with plus signs indicate residual connections. Conv, convolution; BN, batch normalization; AVG Pool, average pooling; CLS class (token)

Our deep-learning (DL) model architecture (Fig. 2) was based on the TransMed model [16], which uses a ResNet [17] to encode slices individually into tokens fed into an encoder-only transformer. For our approach, the ResNet18 backbone was modified to encode each slice into a token while incorporating contextual information from adjacent slices. Residual 3D blocks were integrated between the standard layers of the pre-trained ResNet18 backbone and positioned after the initial max pooling layer and within each basic residual block. This modification preserved the original ResNet18 architecture while utilizing high-quality pre-trained ImageNet [15] weights. Each residual 3D block consisted of a 3 x 3 x 3 convolution, followed by 3D batch normalization and a hyperbolic tangent (Tanh) activation function. By initializing the residual 3D blocks to output zero, the model gradually incorporated 3D information during training, resulting in one 3D-enhanced token per slice. Subsequently, learnable positional embeddings were added, and a class token was prepended. These tokens were fed into an encoder-only transformer [18] for classification. Supplementary Text 1 provides further details on the model architecture and training. Our code is available at: https://github.com/spacecheck/Res3DKneeModel.

### Diagnostic assistance

To assess the model’s potential for diagnostic assistance, we conducted a reader study from September 2024 to January 2025 using 50 MRI studies from the external dataset. Stratified random sampling was employed to ensure sufficient inclusion of rare conditions. Reference standard labels for this dataset were defined through re-reading by two experienced musculoskeletal radiologists (M.S. and S.N., with 6 and 12 years of experience) in consensus. Preliminary findings on model performance did not warrant the inclusion of expert radiologists, so only resident radiologists (*n* = 4) were recruited: two inexperienced residents in their first year of radiology training who had no experience reading knee MRI studies and two experienced residents in their final year who had completed basic MRI training and had read at least 70 knee MRI studies under supervision. The MRI studies were read on in-house radiology workstations, and participants were kept blind to the reference standard findings throughout the whole study.

Two reading sessions were held with a four-week washout to mitigate recall bias. Dedicated reporting templates were designed (Supplementary Fig. 1) using a data privacy law-compliant online survey tool (s2survey.net (SoSci Survey GmbH)). After a broad introduction to the study emphasizing that the AI model was under development, each knee MRI study (in the PACS (isite, Philips)) was presented with the corresponding reporting template (in the survey tool) and demographic data, i.e., age and sex, but no clinical context. In the first (unassisted) session, readers independently reported whether the selected conditions were present or absent. In the second (assisted) session, the MRI studies were re-read in a different order. AI assistance was provided through prefilled binarized model predictions indicating each condition’s presence (yes) or absence (no). Participants could either accept these AI-generated selections or modify them as seen fit. Additionally, a tricolor coding system was used to indicate model performance for each condition based on the full external test set. Model performance for each condition was categorized based on the area under the receiver operating characteristic curve (AUC) as high (green, AUC > 0.85), moderate (yellow, 0.75 ≤ AUC ≤ 0.85), or low (red, AUC < 0.75) (Table 2).

**Table 2:** Details of the deep-learning model’s performance as a function of condition and test set

Condition	Internal dataset	External dataset	Color code	*p*-value
St. p. ACL reconstruction	0.99 (0.99, 1.00)	1.00 (0.99, 1.00)	Green	0.43
MCL tear (partial/complete)	0.95 (0.94, 0.96)	0.88 (0.87, 0.89)	Green	**< 0.001**
Baker’s cyst	0.93 (0.92, 0.94)	0.85 (0.84, 0.87)	Green	**0.001**
Effusion	0.91 (0.90, 0.92)	0.94 (0.93, 0.94)	Green	**0.005**
Lateralization	0.90 (0.88, 0.92)	0.92 (0.90, 0.94)	Green	0.15
ACL tear (partial/complete)	0.89 (0.85, 0.93)	0.89 (0.87, 0.92)	Green	0.80
Medial meniscus extrusion	0.89 (0.88, 0.90)	0.86 (0.84, 0.88)	Green	0.06
Medial meniscus tear	0.88 (0.85, 0.90)	0.83 (0.82, 0.84)	Yellow	**0.006**
Prepatellar bursitis	0.86 (0.81, 0.91)	0.91 (0.81, 1.02)	Green	0.26
Lateral meniscus extrusion	0.85 (0.82, 0.88)	0.73 (0.71, 0.76)	Red	**0.002**
Tibial cartilage pathology	0.85 (0.83, 0.86)	0.74 (0.73, 0.76)	Red	**< 0.001**
Retropatellar cartilage pathology	0.85 (0.82, 0.87)	0.80 (0.78, 0.83)	Yellow	**0.03**
Lateral meniscus tear	0.84 (0.79, 0.89)	0.77 (0.75, 0.79)	Yellow	**0.03**
Femoral cartilage pathology	0.84 (0.83, 0.85)	0.77 (0.76, 0.78)	Yellow	**< 0.001**
Bone marrow edema	0.83 (0.81, 0.86)	0.82 (0.80, 0.84)	Yellow	0.45
Patella alta	0.83 (0.80, 0.87)	0.78 (0.72, 0.83)	Yellow	**0.007**
ACL pathology (non-tear)	0.77 (0.72, 0.82)	0.69 (0.66, 0.71)	Red	**0.03**
Lateral meniscus degeneration	0.76 (0.74, 0.77)	0.74 (0.72, 0.75)	Red	**0.04**
MCL pathology (non-tear)	0.72 (0.69, 0.76)	0.67 (0.63, 0.71)	Red	0.07
LCL pathology (non-tear)	0.72 (0.67, 0.77)	0.58 (0.41, 0.76)	Red	0.15
LCL tear (partial/complete)	0.64 (0.53, 0.75)	0.60 (0.54, 0.65)	Red	0.40
Medial meniscus degeneration	0.61 (0.60, 0.62)	0.58 (0.54, 0.62)	Red	0.25
PCL pathology (tear & non-tear)	0.57 (0.51, 0.64)	0.64 (0.57, 0.72)	Red	0.12

AUC (area under the receiver operating characteristic curve) values are indicated as means (95% confidence interval). Model performance was evaluated based on five-fold cross-validation for the internal dataset, while the entire external dataset was used as the test set. Paired *t*-tests were used to compare the AUC values between internal and external datasets for each condition; significant differences are indicated in bold type. Conditions are ordered according to their AUC values in the internal dataset. Tricolor codes categorize model performance per condition as high (green, AUC > 0.85), moderate (yellow, 0.75 ≤ AUC ≤ 0.85), and low (red, AUC < 0.75). Sensitivities and specificities are provided in Supplementary Table S5

Abbreviations as in Table 1

### Model evaluation and statistical analysis

Statistical analyses were performed by R.V. using Python (v3.10.14) and its libraries pandas (v2.2.1), scikit-learn (v1.3.0), scipy (v1.12.0), and statsmodels (v0.14.4). Model performance was averaged across five cross-validation runs and quantified for each condition using AUC, sensitivity, and specificity. The *t*-distribution was used to compute 95% confidence intervals. Sensitivity and specificity thresholds were determined using Youden’s *J* statistic [19]. AUC values between datasets were compared using paired *t*-tests.

For the reader study, accuracy, sensitivity, specificity, diagnostic efficiency, and inter-reader agreement (quantified by Cohen’s *κ*) were averaged for all MRI studies within each experience level and session and compared between sessions using paired *t*-tests. *p*-values < 0.05 were considered statistically significant.

## Results

The internal data set consisted of 3121 MRI studies from 3018 patients with a mean age of 45 ± 16 years; 51% (*n* = 1524) were female. These studies were requested for post-trauma evaluation (26%), non-traumatic evaluation (55%), and post-surgical evaluation (19%). The external dataset included 458 MRI studies from 429 patients with a mean age of 43 ± 16 years; 45% (*n* = 192) were female. Indications for these studies were post-trauma evaluation (53%), non-traumatic evaluation (33%), and post-surgical evaluation (14%).

**Fig. 3:**
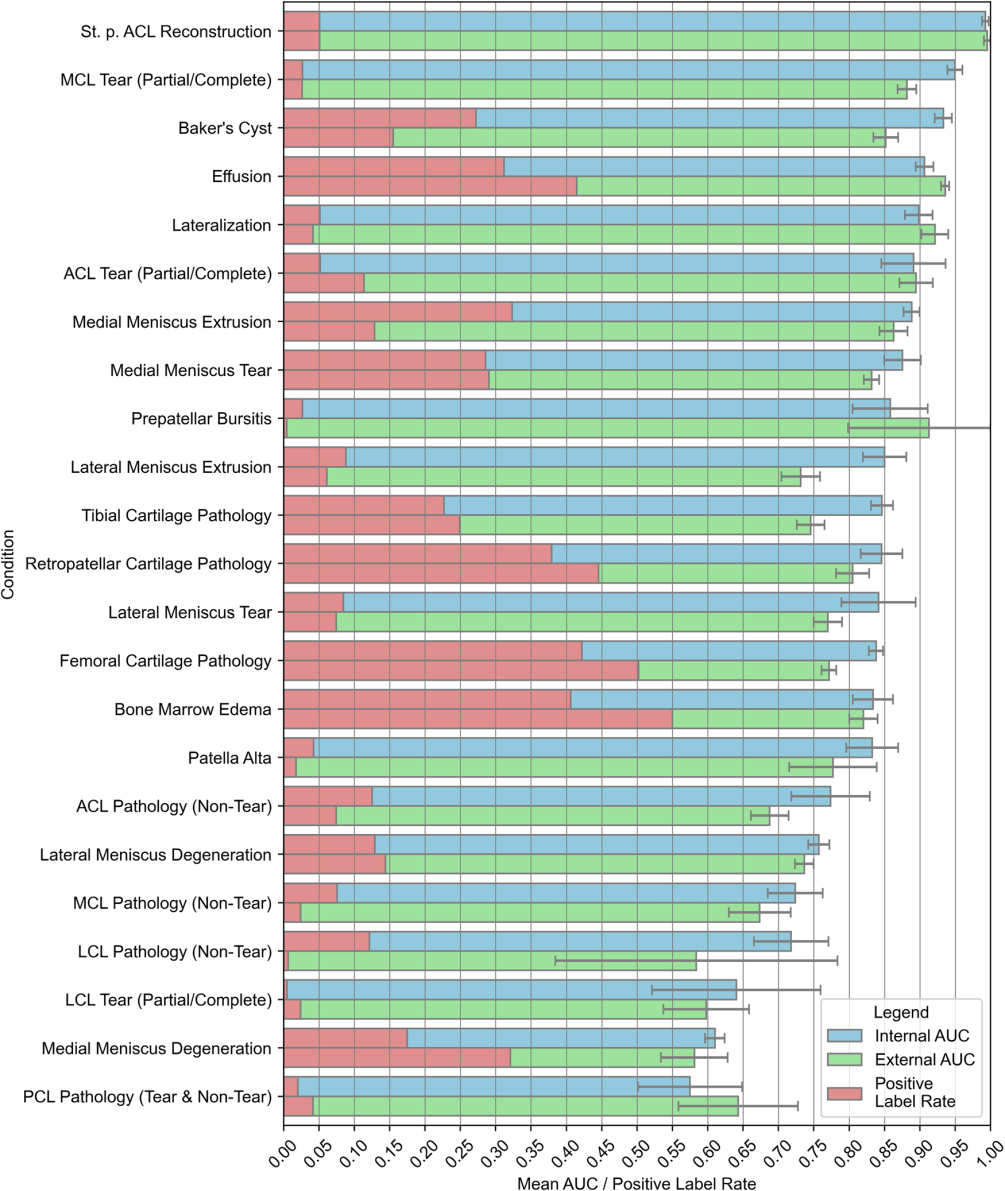
Deep-learning model performance in detecting various knee conditions as a function of dataset and positive label rate. The internal dataset (blue) and the external dataset (green) are indicated. Bars show the area under each condition’s receiver operating characteristic curve (AUC), with error bars representing the 95% confidence interval. The percentage of positive labels in the respective datasets is indicated in red

Figure 3 visualizes the model’s performance as a function of dataset, condition, and positive label rate. In the internal dataset, the model demonstrated robust and largely good-to-excellent performance in identifying most conditions (Table 2). The median AUC was 0.85 (range, 0.57–0.99), the median sensitivity was 76% (range, 27%–97%), and the median specificity was 81% (range, 57%–99%). High, moderate, and low performance were noted for ten, eight, and five conditions, respectively.

These findings were largely reflected in the external test set (Table 2). The median AUC was 0.78 (range, 0.57–0.99), the median sensitivity was 70% (range, 13%–94%), and the median specificity was 80% (range, 48%–99%). High, moderate, and low performance was found in eight, six, and nine conditions, respectively. Supplementary Table 5 details these metrics as a function of dataset and condition.

Despite differences in patient demographics, clinical settings, and image acquisition parameters, the model’s performance remained largely stable between the internal and external datasets, with a mean absolute difference in AUC values of 0.05 ± 0.03. For 11 of the 23 conditions, there were no statistically significant differences in AUC values. Except for effusion, AUC values were lower in the external than the internal dataset.

**Fig. 4:**
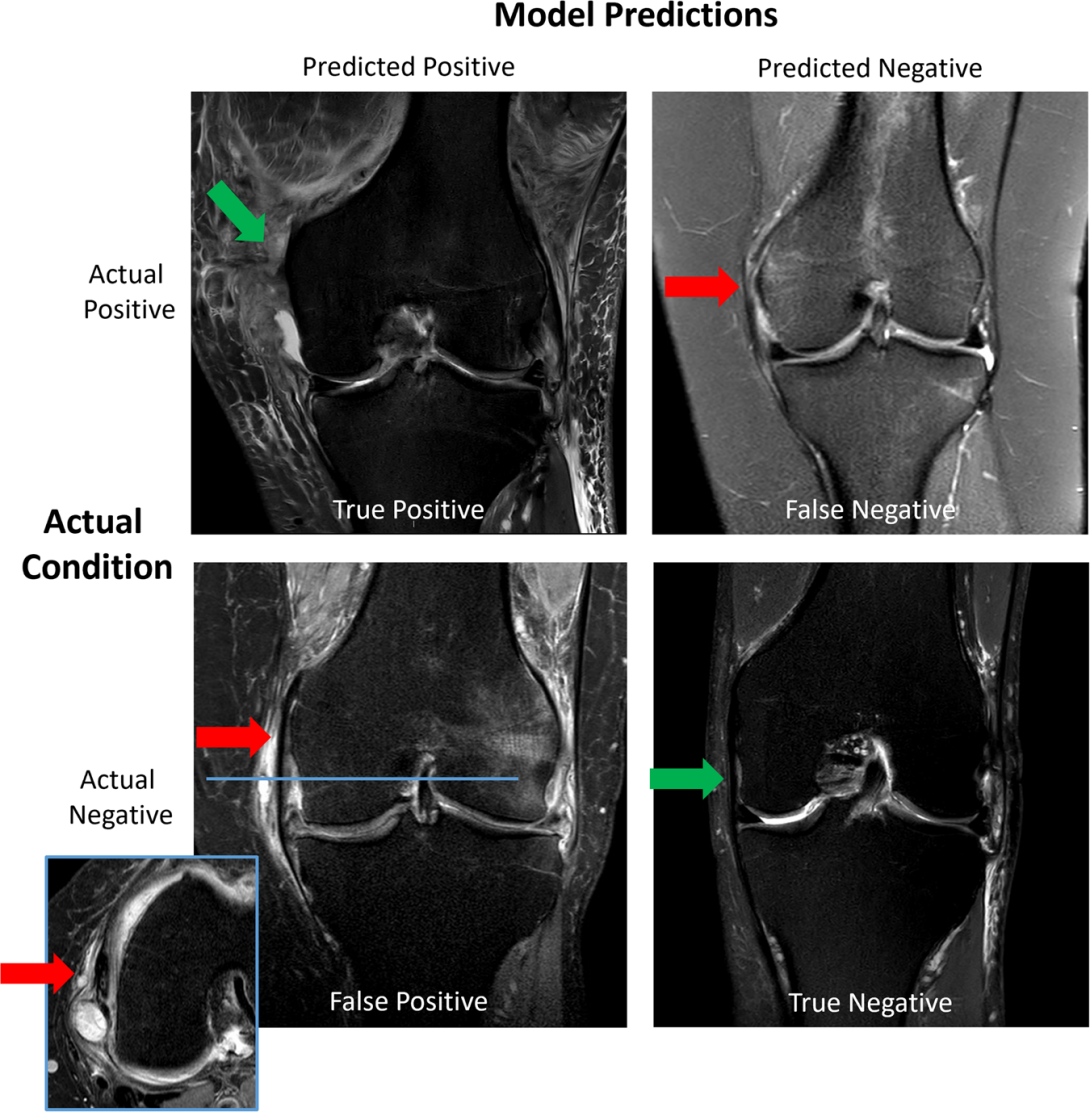
Model predictions vs. ground truth for *MCL Tear (Partial/Complete)*. Possible prediction outcomes are organized in columns, and the ground truth assessments by musculoskeletal radiologists are presented in rows: **True Positive**: The model correctly identified the presence of an MCL tear. Complete femoral avulsion of the MCL (green arrow), with the proximal ligament structure no longer discernible, while the distal ligament portion remains visible. Numerous other ligament injuries, including the cruciate ligaments, occur after knee joint dislocation. **True Negative**: The model correctly identified the absence of an MCL tear (green arrow). **False Negative**: The model failed to identify the MCL tear when it was present. The model missed a partial MCL tear at the femoral insertion (red arrow). **False Positive**: The model incorrectly predicted the presence of an MCL tear when it was absent (red arrows). Edema and ganglion cysts in the periligamentous soft tissues, but structurally intact MCL. Blue vertical line in coronal slice indicates the corresponding axial plane (inset box)

**Fig. 5:**
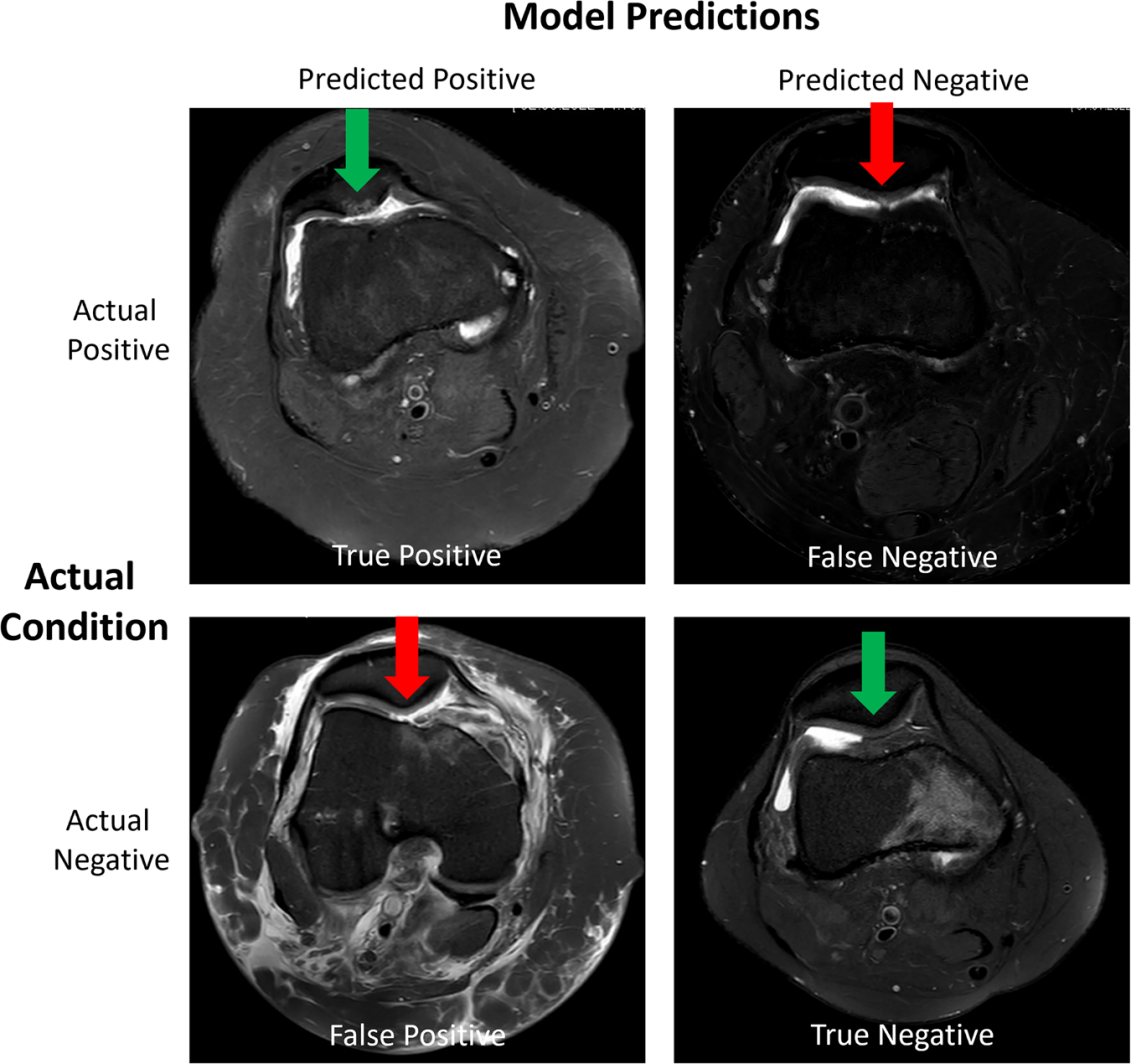
Model predictions vs. ground truth for *Retropatellar Cartilage Pathology*. Figure organization as in Fig. 4. **True Positive**: The model correctly identified the presence of retropatellar cartilage pathology. Substantial full-thickness cartilage defect of the patellar ridge extends into the adjacent medial and lateral patellar facets and the subchondral bone, with reactive bone marrow edema (green arrow). Synovial proliferations and joint effusion indicate chronicity. **True Negative**: The model correctly identified the absence of retropatellar cartilage pathology, regular zonal architecture, and no defect (green arrow). **False Negative**: The model failed to identify retropatellar cartilage pathology when it was present. Small partial-thickness defect of the patellar ridge, without involvement of the subchondral bone. Significant image blurring due to motion artifacts. **False Positive**: The model incorrectly predicted the presence of retropatellar cartilage pathology when there was none. Extensive structural damage following knee joint dislocation, including patellar dislocation: rupture of the medial patellofemoral ligament and the cruciate ligaments, osteochondral defect and fracture of the medial trochlear facet, and pronounced soft tissue edema

**Fig. 6:**
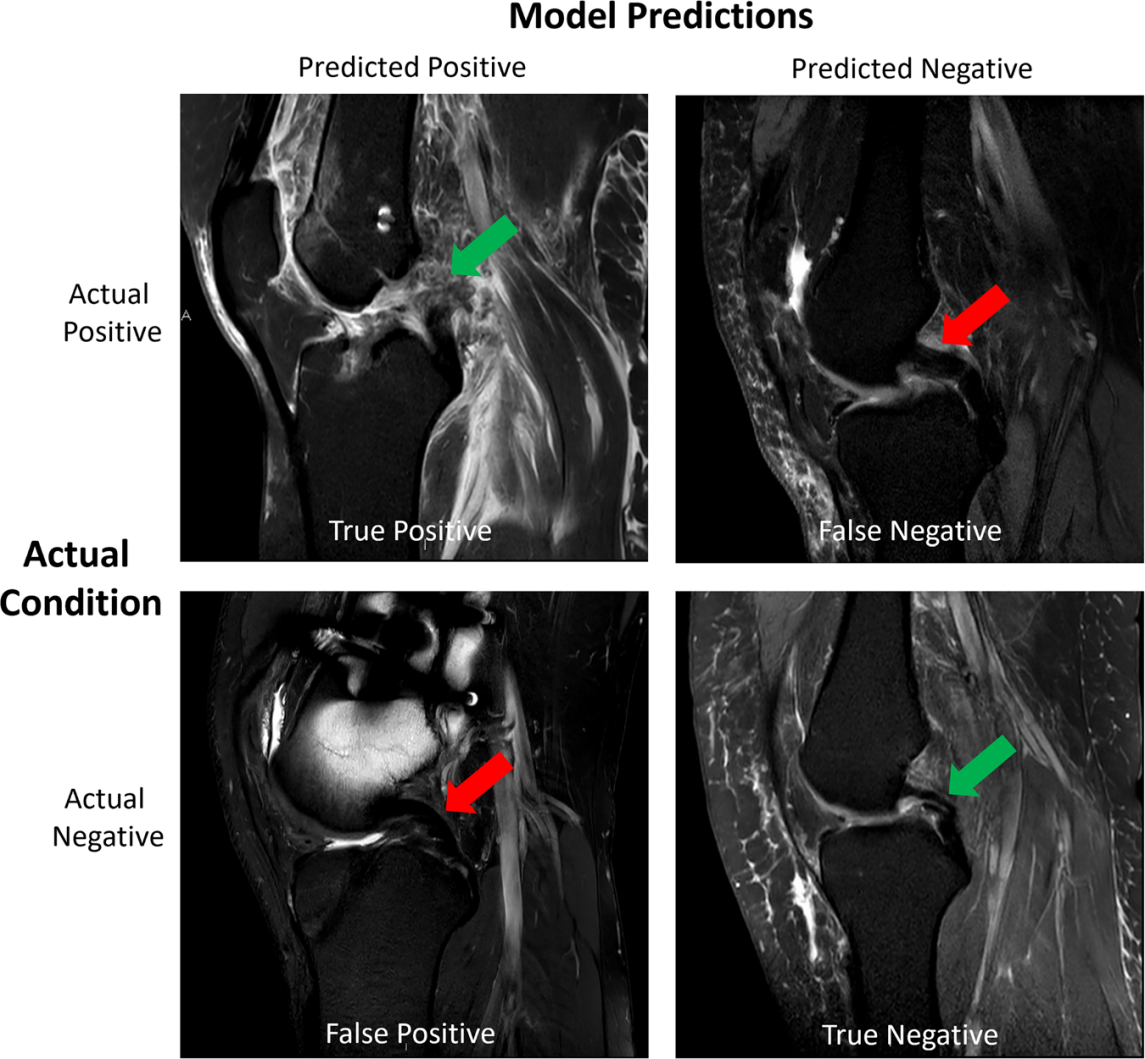
Model predictions vs. ground truth for *PCL Pathology (Tear & Non-Tear)*. Figure organization as in Fig. 4. **True Positive**: The model correctly identified the presence of a PCL tear (as a PCL pathology). Complete femoral avulsion and tearing of the PCL, extending into the ligament’s mid-substance, with fraying of the distal ligament portion (green arrow) and adjacent intra- and periarticular soft tissue edema. **True Negative**: The model correctly identified the absence of PCL pathology despite a thick anterior meniscofemoral ligament anterior to the structurally intact and low-signal PCL (green arrow). **False Negative**: The model failed to identify PCL pathology when it was present. Subtle signal alterations in the proximal third of the PCL (red arrow), possibly degenerative. **False Positive**: The model incorrectly predicted the presence of PCL pathology when there was none, as the PCL was structurally intact. Pronounced susceptibility artifacts secondary to metallic hardware in the femur

Figures 4, 5, and 6 provide example images for qualitative insights on the three different performance categories: *MCL Tear (Partial/Complete)*, (high performance), *Retropatellar Cartilage Pathology*, (moderate performance), and *PCL Pathology (Tear & Non-Tear)*, (low performance). Supplementary Figs. 2 and 3 provide additional examples of *ACL Tear (Partial/Complete)*, and *Medial Meniscus Tear*.

In the reader study, the model’s standalone performance fell between that of inexperienced and experienced residents and influenced those two groups differently (Table 3). When considering all conditions, inexperienced and experienced residents demonstrated significantly increased sensitivity and decreased specificity with model assistance. Additionally, inexperienced residents showed a significant improvement in accuracy. For conditions where the model’s predictions were more reliable (AUC ≥ 0.75), inexperienced residents experienced significant improvements in accuracy, sensitivity, and specificity. Experienced residents showed a significant improvement in sensitivity, a non-significant increase in accuracy, and a marginal (non-significant) decrease in specificity. Without model assistance, experienced residents exhibited higher inter-rater agreement than inexperienced residents. However, model assistance significantly improved inter-rater agreement for both groups, resulting in comparable levels.

**Table 3:** Performance metrics for the deep-learning model and radiology residents as a function of model assistance and condition selection

		DL model	Inexperienced (unassisted)	Inexperienced (assisted)	*p*-value	Experienced (unassisted)	Experienced (assisted)	*p*-value
All conditions	Accuracy	0.79	0.72	0.76	**0.02**	0.82	0.82	0.53
	Sensitivity (%)	68	53	73	**< 0.001**	72	78	**0.002**
	Specificity (%)	79	78	74	**0.02**	84	81	**0.003**
	Agreement (Cohen’s *κ*)	NA	0.17	0.53	**< 0.001**	0.37	0.52	**< 0.001**
Conditions with high and moderate performance	Accuracy	0.86	0.76	0.84	**< 0.001**	0.87	0.88	0.32
	Sensitivity (%)	73	56	75	**< 0.001**	80	84	**0.02**
	Specificity (%)	88	82	85	**0.047**	88	87	0.80
	Agreement (Cohen’s *κ*)	NA	0.25	0.64	**< 0.001**	0.52	0.64	**0.006**

Accuracy, sensitivity, specificity, and inter-reader agreement (Cohen’s *κ*) are indicated for the deep-learning (DL) model and radiology residents—inexperienced and experienced—when reading knee MRI studies without and with DL model assistance. Performance metrics are presented for all conditions and those with high and moderate performance (i.e., AUC ≥ 0.75 (external)). Within each experience level, performance metrics were compared between unassisted and assisted readings using paired *t*-tests; corresponding *p*-values are provided, and significant differences are highlighted in bold type

*NA* not applicable, *DL* deep learning

Consistent with these findings, inexperienced residents more often accepted alternative model suggestions, thereby improving their diagnostic accuracy (Table 4). In contrast, experienced residents changed their initial assessments less frequently and encountered fewer discrepancies relative to the model’s suggestions.

**Table 4:** Model impact on the diagnostic assessments of radiology residents as a function of resident experience and model performance

Resident experience	Model performance	Resident vs. AI discrepancy (%)	Changes accepted (%)	Correct changes (%)
Inexperienced	Low	41	78	50
	Moderate	32	80	75
	High	25	85	71
Experienced	Low	33	52	47
	Moderate	25	51	61
	High	15	57	61

Resident vs. AI discrepancy (%) indicates the percentage of individual conditions for which the residents’ initial (unassisted) assessment differed from the model output (artificial intelligence, AI)

Changes accepted (%) indicates the percentage of these discrepant assessments where residents changed their initial assessment to match the model output

Correct changes (%) indicate the percentage of accepted changes that were correct relative to the reference standard

Model performance is stratified according to each condition’s area under the receiver operating characteristic curve (AUC) value, with *low* indicating AUC < 0.75, *moderate* indicating 0.75 ≤ AUC ≤ 0.85, and *high* indicating AUC > 0.85

Both groups were more likely to accept alternative suggestions when the model’s performance was higher. However, for low-performance conditions, the relatively high rates of accepted changes (52% (experienced) and 78% (inexperienced)) were associated with diagnostic improvements close to chance (47% and 50%), indicating limited benefit from less reliable model outputs but no substantial decline in accuracy. Mean reading times per study decreased by 4% for inexperienced residents (3.5 to 3.4 min; *p* = 0.28) and 10% for experienced residents (4.7 to 4.2 min; *p* = 0.045).

## Discussion

In this study, we developed a deep-learning model for automated multi-condition analysis of routine knee MRI studies and evaluated its clinical utility for assisting resident radiologists. The model demonstrated solid performance, achieving moderate or high AUCs (≥ 0.75) for 18 of 23 conditions on the internal dataset. It generalized well to an external dataset with a mean absolute AUC difference of 0.05 ± 0.03. Model assistance significantly increased sensitivity and inter-reader agreement between residents and shortened reading times for experienced residents. While a significant decrease in specificity was observed for inexperienced and experienced residents when considering all conditions, this was no longer the case when considering moderate-to-high-performing conditions (AUC ≥ 0.75) only. In this setting, inexperienced residents showed significant improvements in accuracy, sensitivity, and specificity, while experienced residents improved in sensitivity and had no significant decreases in accuracy and specificity, making model assistance strictly beneficial.

Model performance was influenced by architecture, data quality, and morphological diversity. We based our model on an established multi-sequence framework [16], enhancing it with residual 3D blocks for high-resolution contextual information. Regularization strategies such as ImageNet initialization and shared slice encoders across sequences helped mitigate overfitting and performance drops across datasets. The MRI studies and report-based annotations dataset, used for training, was sourced from a high-volume radiologic practice. Such reports are not always a high-quality standard of reference [20], as they are primarily intended for clinical communication. To balance quality and quantity, trained pre-graduate medical students conducted initial labeling, with board-certified radiologists ensuring annotation quality. Still, some conditions, such as those affecting the PCL, LCL, or prepatellar bursa, are rare, resulting in small sample sizes. To maintain sufficient sample sizes in our study, we adopted broader pathological definitions, simplifying classifications to binary (PCL) and ternary (MCL, LCL) categories.

Morphologic diversity further impacted model performance. Cartilage pathologies, bone marrow edema, meniscus degeneration, or non-tear ligament pathologies exhibit subtle imaging features, lack unequivocal diagnostic criteria, or rely on subjective grading, resulting in relatively low inter-reader agreement [21, 22]. For these conditions, differentiating pathologic changes from physiologic (age-related) changes is challenging, especially for DL models that interpret MRI studies without context, such as the patient’s history or physical findings. In contrast, St. p. ACL reconstruction, Baker’s cyst, effusion, prepatellar bursitis, and meniscus tears have distinct imaging features and well-established diagnostic criteria.

Compared to previous studies focused on single conditions or tissues [10–13], our model addressed a broader clinical spectrum. While direct comparisons are limited by varying methods and data, our model performed largely on par with published benchmarks. Liu et al [23] and Xue et al [24] reported high AUCs (0.98–0.99) for complete ACL tears using detailed annotations like slice-level labels or ACL segmentations. Our model achieved AUCs of 0.89 for partial and complete ACL tears without such annotations. Similarly, our model’s AUCs for medial meniscus tears (0.88 (internal), 0.83 (external)) and lateral meniscus tears (0.84, 0.77) are comparable to those reported by others [25, 26]. While incorporating segmentation and regions of interest may enhance model performance, we opted for a simpler and less condition-specific approach, processing labels uniformly and eliminating the need for additional extensive labeling.

Developing the only other many-condition model published, Astuto et al [11] built a hierarchical processing pipeline relying on multi-tissue segmentation to qualitatively assess cartilage, menisci, the ACL, and bone marrow. Their model performed similarly to ours for bone marrow edema (AUC 0.83 vs. 0.82 (external)), though other comparisons are limited by differing groupings and definitions of conditions. Unlike their research-oriented approach based on a single 3D sequence, our model was designed for routine clinical protocols.

Beyond research settings, Keros (Incepto) is currently the only CE-certified AI tool for diagnostic assistance in knee MRI. It automatically detects ACL and MCL tears, meniscal and cartilage lesions, bone edema, joint effusions, and popliteal cysts, and provides quantitative measures of patellar height, trochlear morphology, and TT-TG distance [27–29]. In contrast, our model addresses a broader spectrum of 23 knee conditions with transparent, condition-level performance reporting, including challenging areas such as PCL and LCL tears. This comprehensive coverage and transparency aim to provide users with a realistic understanding of strengths and weaknesses, guiding targeted model refinement in the future.

Our study has several limitations. The current DL model classifies 23 knee conditions using binary (or in some cases ternary) decisions, which do not reflect the nuanced severity gradations and morphometric details that are often required for clinical decision-making. Certain conditions (e.g., PCL, LCL, prepatellar bursa lesions) were rare, and broader pathological definitions were therefore applied, which reduced diagnostic granularity. Training data were derived from a single high-volume center and were based largely on routine clinical reports and radiologist reads rather than on repeated expert consensus or arthroscopic confirmation. This could have introduced systematic labeling errors that compromise both model accuracy and its assistance capabilities, despite high stand-alone performance metrics. Moreover, reliance on a single-center dataset may have introduced bias, as reflected by the observed decrease in performance from internal to external testing for certain conditions. Furthermore, both datasets consisted exclusively of conventionally acquired images, leaving the model’s performance on AI-accelerated MRI protocols untested.

Additionally, the generalizability of our findings is limited by aspects of our reader study. The study involved only a small number of residents, excluding board-certified specialists, which limits the scope of the clinical validation. We focused on residents because they were expected to benefit most from AI assistance, and the model’s stand-alone performance, which fell between that of junior and senior residents, was unlikely to improve expert accuracy. This choice also avoided unnecessary demands on experts’ time. Nevertheless, the impact on expert MSK radiologists remains unknown, and prior prospective work in neuroradiology (and the detection of intracranial hemorrhage) has shown that specialized radiologists may not benefit from AI assistance [30]. Moreover, to maintain a fair comparison with the model, which did not have access to a broader clinical context, we withheld referral information and other pertinent clinical details from the readers. While this reduced case memorability for readers, it may have affected their diagnostic performance. Beyond model accuracy, the human-AI interaction interface will be critical for real-world deployment, particularly as more advanced AI models convey more nuanced diagnostic information. In our study, model predictions were displayed via a simple checkbox interface. The question of which approach conveys AI outputs effectively, safely, and without distracting the reader is yet unanswered, and various delivery methods, such as pre-populated report templates, integrated visualizations, or image annotations, need to be evaluated. Future studies should also consider incorporating larger and more experienced groups of readers, including subspecialty-trained MSK radiologists, as well as a richer clinical context, to evaluate the clinical utility of the model better.

In conclusion, our DL model demonstrated solid performance and generalizability for numerous relevant knee conditions, enhancing sensitivity, inter-reader agreement, and efficiency—especially for inexperienced residents. However, reduced specificity and hints of overreliance on model outputs warrant cautious implementation. Future research should expand condition coverage, refine grading, and assess real-world workflow compatibility.

## Supplementary information

Supplementary Material

## Abbreviations

ACL: Anterior cruciate ligament
AUC: Area under the receiver operating characteristic curve
DL: Deep learning
LCL: Lateral cruciate ligament
MCL: Medial cruciate ligament
PCL: Posterior cruciate ligament
