# Supplementary material for: Comprehensive deep learning-assisted multi-condition analysis of knee MRI studies improves resident radiologist performance
Source: Eur Radiol. 2025 Oct 17;36(4):2563–75. doi: 10.1007/s00330-025-12052-8 (PMC13035746; doi:10.1007/s00330-025-12052-8)
Supplement: Supplementary file 1 — Supplementary Material [file 330_2025_12052_MOESM1_ESM.pdf]

Supplementary Material

Title: Comprehensive Deep Learning-Assisted Multi-Condition Analysis of Knee MRI Studies Improves Resident Radiologist Performance

Supplementary Table 1: Image Acquisition Details.
Sequence parameters are presented as means ± standard deviation (where appropriate). Proton Density-weighted fat-saturated (PD fs) sequences were acquired in the three principal orientations, i.e., sagittal, coronal, and axial, while T1-weighted sequences were acquired in the sagittal orientation. All sequences were acquired without AI-accelerated image acquisition protocols.

		**Internal Dataset**		**External Dataset**			
	**Manufacturer, Scanner, Field Strength**	Philips Intera 1.5 T	Siemens Magnetom Skyra 3.0 T	Philips Ingenia Elition X 3 T	Philips Ingenia Ambition X 1.5 T	Philips Achieva 3.0 T	Philips Ingenia 1.5 T
	**Coil channels [n]**	8	15	16	8	16	8
	**Patients [n]**	3025	96	155	160	74	69
**PD fs**	**Repetition Time [ms]**	3309 ± 741	3791 ± 320	3076 ± 235	4058 ± 404	5432 ± 712	4124 ± 219
	**Echo Time [ms]**	21 ± 4	38 ± 2	40 ± 1	29 ± 1	30 ± 0	30 ± 0
	**Flip Angle [°]**	90	180	90	90	90	90
	**Acquired Pixel Resolution [mm/pixel]**	0.35 ± 0.03	0.34 ± 0.07	0.22 ± 0.02	0.30 ± 0.03	0.32 ± 0.01	0.30 ± 0.01
	**Slice Thickness [mm]**	3.0 ± 0.1	3.1 ± 0.2	2.7 ± 0.2	3.0 ± 0.0	3.0 ± 0.0	3.0 ± 0.0
	**Slices [n]**	32 ± 17	31 ± 5	45 ± 4	34 ± 3	35 ± 3	34 ± 3
	**Number of Signal Averages**	2 ± 1	1 ± 0	2 ± 0	2 ± 0	1 ± 0	2 ± 0
	**Turbo Factor**	15 ± 4	7 ± 1	11 ± 1	15 ± 4	14 ± 1	13 ± 1
**T1**	**Repetition Time [ms]**	579 ± 104	596 ± 117	658 ± 36	474 ± 40	624 ± 84	469 ± 34
	**Echo Time [ms]**	15 ± 4	22 ± 4	15 ± 0	10 ± 0	9 ± 0	10 ± 0
	**Flip Angle [°]**	90	180	90	90	90	90
	**Acquired Pixel Resolution [mm/pixel]**	0.36 ± 0.04	0.29 ± 0.05	0.26 ± 0.01	0.25 ± 0.01	0.36 ± 0.01	0.25 ± 0.01
	**Slice Thickness [mm]**	3.0 ± 0.0	3.0 ± 0.1	2.5 ± 0.0	3.0 ± 0.0	3.0 ± 0.0	3.0 ± 0.0
	**Slices [n]**	24 ± 2	30 ± 1	44 ± 3	32 ± 2	33 ± 2	33 ± 2
	**Number of Signal Averages**	2 ± 0	1 ± 0	2 ± 0	1 ± 0	1 ± 0	1 ± 0
	**Turbo Factor**	4 ± 1	5 ± 0	5 ± 0	4 ± 0	3 ± 0	4 ± 0

Supplementary Table 2: Diagnostic Criteria of Knee Joint Conditions.

"Essential Criteria" were necessary for diagnosing the specific condition, while "Supporting Criteria" strengthened the diagnosis but were not mandatory. Unless specified otherwise, signal intensities refer to T2-weighted or Proton Density (PD)-weighted images.

Condition	Essential Criteria	Supporting Criteria
**St. p. ACL Reconstruction**	Presence of ACL graft material on MRI	Surgical tunnels in tibia and femur<br>Surgical hardware (e.g., screws, fixation devices) around tibia and femur
**MCL Tear (Partial/Complete)**	Disruption or discontinuity of MCL fibers	High signal within and around MCL
**Baker's Cyst**	Fluid-filled cyst (between medial head of gastrocnemius and semimembranosus tendon)	Continuity with joint space<br>Thin cyst wall
**Effusion**	Excess intra-articular fluid within the joint	Distension of the suprapatellar bursa
**Lateralization (of Patella)**	Lateral displacement of patella relative to trochlea (TT-TG ≥ 15 mm)	-
**ACL Tear (Partial/Complete)**	Disruption or discontinuity of ACL fibers	Increased signal within ACL<br>Abnormal orientation of ACL<br>Typical bone bruises
**Medial Meniscus Extrusion**	Medial meniscus base displaced ≥ 3 mm beyond tibial margin	-
**Medial Meniscus Tear**	Linear high signal extending to meniscal surface on at least two adjacent slices	Abnormal meniscal morphology
**Prepatellar Bursitis**	Fluid collection within prepatellar bursa	Thickened bursal walls
**Lateral Meniscus Extrusion**	Lateral meniscus base displaced ≥ 3 mm beyond tibial margin	-
**Tibial Cartilage Pathology**	Cartilage thinning or defects on the tibial plateau	High (or low) signal within cartilage tissue
**Retropatellar Cartilage Pathology**	Cartilage thinning or defects on retropatellar surface	High (or low) signal within cartilage tissue
**Lateral Meniscus Tear**	Linear high signal extending to meniscal surface on at least two adjacent slices	Abnormal meniscal morphology
**Femoral Cartilage Pathology**	Cartilage thinning or defects on femoral condyles	High (or low) signal within cartilage tissue
**Bone Marrow Edema**	Increased signal within bone marrow on T2-weighted or PD-weighted images	Decreased signal within bone marrow on corresponding T1-weighted images
**Patella Alta**	Caton-Deschamps Index > 1.3	-
**ACL Pathology (Non-Tear)**	Abnormal signal within intact ACL	Thickened ACL without fiber disruption (mucoid degeneration)<br>Ganglion cysts adjacent to ACL<br>St. p. resorption
**Lateral Meniscus Degeneration**	Increased intrameniscal signal not extending to meniscal surface	Inhomogeneous meniscal texture
**MCL Pathology (Non-Tear)**	Thickened MCL with increased signal	Periligamentous edema<br>Intraligamentous calcifications
**LCL Pathology (Non-Tear)**	Thickened LCL with increased signal	Periligamentous edema<br>Degenerative changes without fiber disruption
**LCL Tear (Partial/Complete)**	Disruption or discontinuity of LCL fibers	High signal intensity within and around LCL
**Medial Meniscus Degeneration**	Increased intrameniscal signal not extending to meniscal surface	Inhomogeneous meniscal texture

**PCL Pathology (Tear & Non-Tear)**	For Tear: Disruption, discontinuity, or abnormal signal of PCL fibers<br>For Non-Tear: Thickened PCL with increased signal but intact fibers	For Tear: Abnormal PCL contour, typical bone bruises

Supplementary Table 3: Dataset Characteristics and Condition Counts for Internal Dataset Folds and the External Test Set.

Except for age, which is indicated as mean ± standard deviation, values are numbers of positive counts (percentages).

Characteristic	Fold #1	Fold #2	Fold #3	Fold #4	Fold #5	External
**Patient Demographics**						
Unique Patients [n]	597	613	597	602	609	429
Age [years]	44 ± 16	45 ± 15	44 ± 15	45 ± 16	45 ± 16	43 ± 16
Female	307 (51.4)	300 (48.9)	297 (49.8)	312 (51.8)	308 (50.6)	192 (44.8)
Male	290 (48.6)	313 (51.1)	300 (50.2)	290 (48.2)	301 (49.4)	237 (55.3)
**MRI Studies [n]**	626	631	617	615	632	458
St. p. ACL Reconstruction	31 (5.0)	33 (5.2)	32 (5.2)	32 (5.2)	31 (4.9)	36 (7.9)
MCL Tear (Partial/Complete)	16 (2.6)	16 (2.5)	19 (3.1)	16 (2.6)	16 (2.5)	12 (2.6)
Baker's Cyst	171 (27.3)	170 (26.9)	170 (27.6)	169 (27.5)	169 (26.7)	71 (15.5)
Effusion	194 (31.0)	196 (31.1)	195 (31.6)	194 (31.5)	194 (30.7)	190 (41.5)
Lateralization	32 (5.1)	32 (5.1)	32 (5.2)	32 (5.2)	32 (5.1)	19 (4.1)
ACL Tear (Partial/Complete)	32 (5.1)	34 (5.4)	32 (5.2)	32 (5.2)	32 (5.1)	52 (11.4)
Medial Meniscus Extrusion	202 (32.3)	201 (31.9)	204 (33.1)	201 (32.7)	201 (31.8)	59 (12.9)
Medial Meniscus Tear	178 (28.4)	179 (28.4)	179 (29.0)	178 (28.9)	178 (28.2)	133 (29.0)
Prepatellar Bursitis	17 (2.7)	16 (2.5)	16 (2.6)	17 (2.8)	17 (2.7)	2 (0.4)
Lateral Meniscus Extrusion	55 (8.8)	55 (8.7)	55 (8.9)	55 (8.9)	55 (8.7)	28 (6.1)
Tibial Cartilage Pathology	141 (22.5)	142 (22.5)	141 (22.9)	141 (22.9)	143 (22.6)	114 (24.9)
Retropatellar Cartilage Pathology	239 (38.2)	236 (37.4)	236 (38.2)	236 (38.4)	236 (37.3)	204 (44.5)
Lateral Meniscus Tear	53 (8.5)	52 (8.2)	53 (8.6)	52 (8.5)	54 (8.5)	34 (7.4)
Femoral Cartilage Pathology	263 (42.0)	264 (41.8)	263 (42.6)	264 (42.9)	263 (41.6)	230 (50.2)
Bone Marrow Edema	254 (40.6)	253 (40.1)	253 (41.0)	254 (41.3)	253 (40.0)	252 (55.0)
Patella Alta	27 (4.3)	26 (4.1)	27 (4.4)	27 (4.4)	26 (4.1)	8 (1.7)
ACL Pathology (Non-Tear)	78 (12.5)	78 (12.4)	78 (12.6)	78 (12.7)	79 (12.5)	34 (7.4)
Lateral Meniscus Degeneration	81 (12.9)	81 (12.8)	81 (13.1)	80 (13.0)	80 (12.7)	66 (14.4)
MCL Pathology (Non-Tear)	48 (7.7)	48 (7.6)	47 (7.6)	47 (7.6)	47 (7.4)	11 (2.4)
LCL Pathology (Non-Tear)	75 (12.0)	76 (12.0)	77 (12.5)	76 (12.4)	75 (11.9)	3 (0.7)
LCL Tear (Partial/Complete)	3 (0.5)	3 (0.5)	3 (0.5)	3 (0.5)	3 (0.5)	11 (2.4)
Medial Meniscus Degeneration	109 (17.4)	109 (17.3)	110 (17.8)	109 (17.7)	109 (17.2)	147 (32.1)
PCL Pathology (Tear & Non-Tear)	12 (1.9)	13 (2.1)	12 (1.9)	12 (2.0)	14 (2.2)	19 (4.1)

Supplementary Table 4: Image Augmentation Strategy.
Parameter details for augmenting each image stack during training.

**Augmentation**	**Parameters**
Resized Crop	x crop: [0.85, 1.0]<br>y crop: [0.85, 1.0]<br>rescale to original size
Rotation	angle range: [-15°, 15°] (in xy plane)
Brightness	Scaling factor range: [0.5, 2.0]
Contrast	Scaling factor range: [0.5, 2.0]
Sharpness	Scaling factor range: [0.0, 2.0]
Addition of Gaussian Noise	Sample from distribution N(0, 0.01)
Blacking Out Individual Slices	Probability: 10%

Supplementary Table 5: Details of the Deep Learning Model's Performance as a Function of Condition, Test Set, and Performance Metric.

For the internal dataset, performance metrics are reported as mean (95% Confidence Interval) for the five-fold cross-validation. For the external dataset, performance metrics are reported for the entire dataset used as the test set. AUC - Area under the receiver operating characteristic curve.

	**Internal Dataset**			**External Dataset**		
**Condition**	**AUC**	**Sensitivity**	**Specificity**	**AUC**	**Sensitivity**	**Specificity**
St. p. ACL Reconstruction	0.99 (0.99, 1.00)	97 (97, 97)	99 (98, 99)	1.00 (0.99, 1.00)	94 (94, 94)	99 (98, 99)
MCL Tear (Partial/Complete)	0.95 (0.94, 0.96)	88 (84, 92)	90 (85, 94)	0.88 (0.87, 0.89)	77 (73, 81)	86 (82, 90)
Baker's Cyst	0.93 (0.92, 0.94)	87 (84, 90)	88 (86, 91)	0.85 (0.84, 0.87)	78 (75, 81)	82 (79, 85)
Effusion	0.91 (0.90, 0.92)	86 (81, 90)	82 (78, 86)	0.94 (0.93, 0.94)	86 (82, 91)	87 (82, 92)
Lateralization	0.90 (0.88, 0.92)	82 (75, 90)	82 (76, 88)	0.92 (0.90, 0.94)	83 (73, 94)	85 (77, 93)
ACL Tear (Partial/Complete)	0.89 (0.85, 0.93)	79 (71, 87)	90 (79, 100)	0.89 (0.87, 0.92)	79 (72, 85)	89 (85, 94)
Medial Meniscus Extrusion	0.89 (0.88, 0.90)	80 (77, 84)	84 (79, 88)	0.86 (0.84, 0.88)	82 (80, 84)	80 (76, 83)
Medial Meniscus Tear	0.88 (0.85, 0.90)	84 (80, 88)	81 (77, 84)	0.83 (0.82, 0.84)	73 (66, 80)	80 (73, 87)
Prepatellar Bursitis	0.86 (0.81, 0.91)	73 (66, 81)	85 (80, 90)	0.91 (0.81, 1.02)	50 (50, 50)	83 (62, 100)
Lateral Meniscus Extrusion	0.85 (0.82, 0.88)	76 (74, 79)	81 (75, 87)	0.73 (0.71, 0.76)	57 (51, 63)	84 (75, 93)
Tibial Cartilage Pathology	0.85 (0.83, 0.86)	77 (74, 80)	77 (72, 82)	0.75 (0.73, 0.76)	68 (65, 72)	70 (64, 75)
Retropatellar Cartilage Pathology	0.85 (0.82, 0.87)	74 (65, 82)	81 (74, 87)	0.80 (0.78, 0.83)	69 (64, 74)	79 (75, 83)
Lateral Meniscus Tear	0.84 (0.79, 0.89)	72 (62, 82)	85 (82, 89)	0.77 (0.75, 0.79)	74 (64, 84)	70 (59, 80)
Femoral Cartilage Pathology	0.84 (0.83, 0.85)	77 (73, 81)	78 (73, 83)	0.77 (0.76, 0.78)	66 (58, 74)	76 (69, 84)
Bone Marrow Edema	0.83 (0.81, 0.86)	69 (60, 78)	85 (79, 92)	0.82 (0.80, 0.84)	70 (63, 76)	83 (80, 86)
Patella Alta	0.83 (0.80, 0.87)	73 (58, 87)	77 (65, 90)	0.78 (0.72, 0.83)	65 (53, 77)	82 (76, 87)
ACL Pathology (Non-Tear)	0.77 (0.72, 0.82)	61 (50, 72)	81 (74, 88)	0.69 (0.66, 0.71)	65 (53, 76)	65 (52, 78)
Lateral Meniscus Degeneration	0.76 (0.74, 0.77)	77 (65, 89)	64 (53, 75)	0.74 (0.72, 0.75)	71 (66, 76)	67 (62, 73)
MCL Pathology (Non-Tear)	0.72 (0.69, 0.76)	63 (51, 75)	71 (58, 85)	0.67 (0.63, 0.71)	76 (59, 93)	48 (30, 66)
LCL Pathology (Non-Tear)	0.72 (0.67, 0.77)	64 (56, 72)	72 (63, 80)	0.58 (0.41, 0.76)	13 (0, 46)	93 (77, 100)
LCL Tear (Partial/Complete)	0.64 (0.53, 0.75)	27 (0, 58)	78 (52, 100)	0.60 (0.54, 0.65)	31 (0, 63)	78 (51, 100)
Medial Meniscus Degeneration	0.61 (0.60, 0.62)	61 (41, 81)	57 (37, 77)	0.58 (0.54, 0.62)	44 (33, 55)	71 (62, 80)
PCL Pathology (Tear & Non-Tear)	0.57 (0.51, 0.64)	46 (24, 67)	71 (52, 89)	0.64 (0.57, 0.72)	52 (31, 72)	70 (54, 85)

Supplementary Text 1: **Details on the Implementation of the 3D Residual Convolutions and Training Details.**

The introduced 3D convolutions aim to enable the model to incorporate high-resolution information from adjacent images early in the computation of the image tokens. Each 3x3x3 convolution is followed by 3D batch normalization and a hyperbolic tangent (Tanh) activation function, constituting the 3D block. This block is implemented as a residual function, so if its output is zero, the model effectively relies solely on the regular 2D ResNet18 layers to encode an image.

To enable the gradual incorporation of 3D information during training, 3D blocks are initialized to output zero at the outset of training. The convolutions are initialized with random weights to allow for effective learning, while the batch normalization layers are initialized with scale (γ) and bias (β) of zero. This initialization strategy ensures that the batch normalization output is zero initially, independent of the input, as described by the batch normalization equation:

$$y = \frac{x - E[x]}{\sqrt{Var[x] + \varepsilon}} * \gamma + \beta$$

As training progresses, the parameters of the 3D block are updated to incorporate 3D information into the pre-trained ResNet18 intermediate representations. Finally, the Tanh activation function is chosen over ReLU after batch normalization because Tanh outputs zero for zero input and is differentiable at zero, mitigating potential gradient flow issues at the start of training.

Training Details

We implemented a standard five-fold cross-validation protocol to evaluate model performance. Each model was trained five times, with a different fold held out as the test set, while the remaining four folds were used for training and validation. Separate models were trained for each condition to account for differences in convergence rates, which mitigated overfitting. Models were trained using stochastic gradient descent with a momentum coefficient of 0.7, weight decay of 0.01, and a learning rate of 0.001. A batch size of 10 was used during training. Early stopping was employed to prevent overfitting, with training terminated if the area under the receiver operating characteristic curve (AUC) on the validation set did not improve for 25 consecutive epochs. To address class imbalance, we applied a weighted binary cross-entropy loss function, where weights were calculated as the ratio of positive to negative samples. During training of a model, the top five model checkpoints with the highest AUC on the validation set were saved. These checkpoints were subsequently used for model ensembling, which reduced variability across training runs and folds.

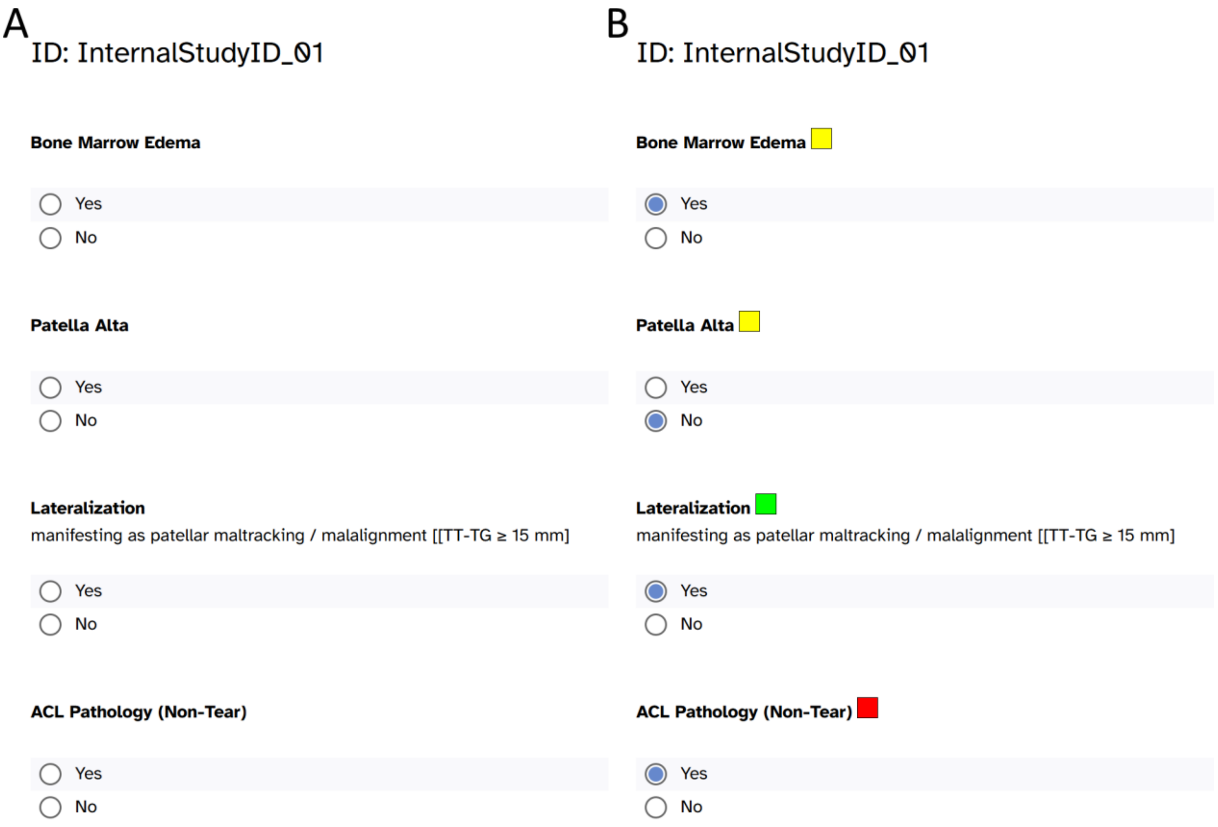

Supplementary Figure 1: Excerpt from survey tool for reader study. MRI studies (n = 50) were read in two sessions – unassisted (A) and assisted by the deep learning model (B). Assistance was provided by prefilling the binarized selection (yes/no) for each condition based on the model outputs as well as indicating the model performance for each condition using a tricolor coding system: red (low performance, AUC < 0.75), yellow (moderate performance, 0.75 ≤ AUC ≤ 0.85), and green (high performance, AUC > 0.85).

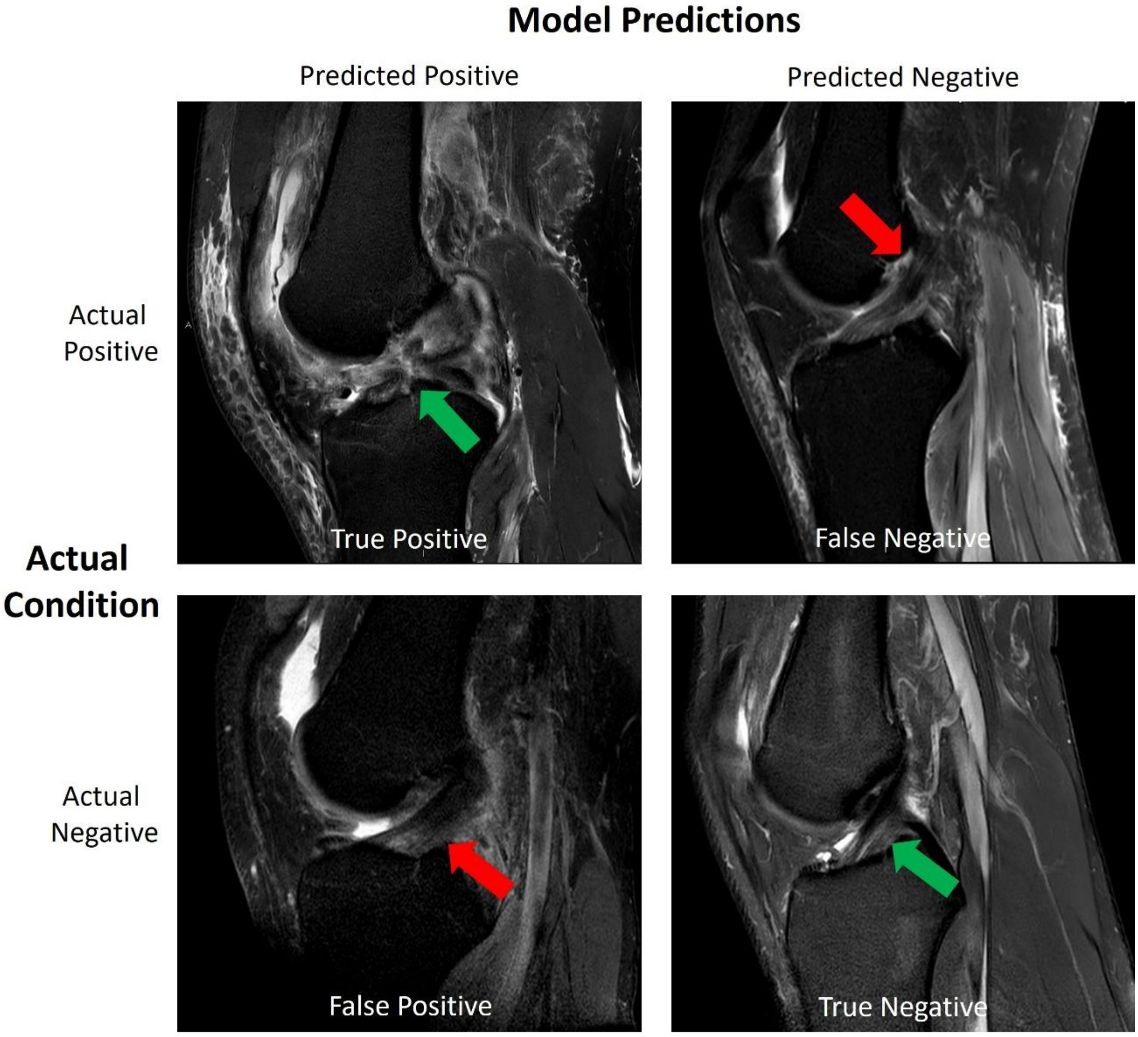

Supplementary Figure 2: Model predictions vs. ground truth for *ACL Tear (Partial/Complete)*. Figure organization as in Figure 4.

- True Positive: The model correctly identified the presence of an ACL tear. Edematous soft tissue in the intercondylar notch, but no ACL discernible (green arrow). Complex multi-ligamentous injury following knee joint dislocation, including injuries to the collateral ligaments, cruciate ligaments, medial meniscus, and popliteus tendon.
- True Negative: The model correctly identified the absence of an ACL tear. ACL with a regular and taut appearance (green arrow).
- False Negative: The model failed to identify an ACL tear when it was present. Moderate swelling, elongation, focal discontinuity of the ACL at the femoral insertion (red arrow), and an abnormally flattened orientation relative to the roof of the notch. The excessive anterior translation of the tibia relative to the femur, indicating functional ACL insufficiency, is not shown.
- False Positive: The model incorrectly predicted the presence of an ACL tear when there was none. Regular appearance of the ACL (red arrow). Not shown is a partial rupture of the posterior cruciate ligament.

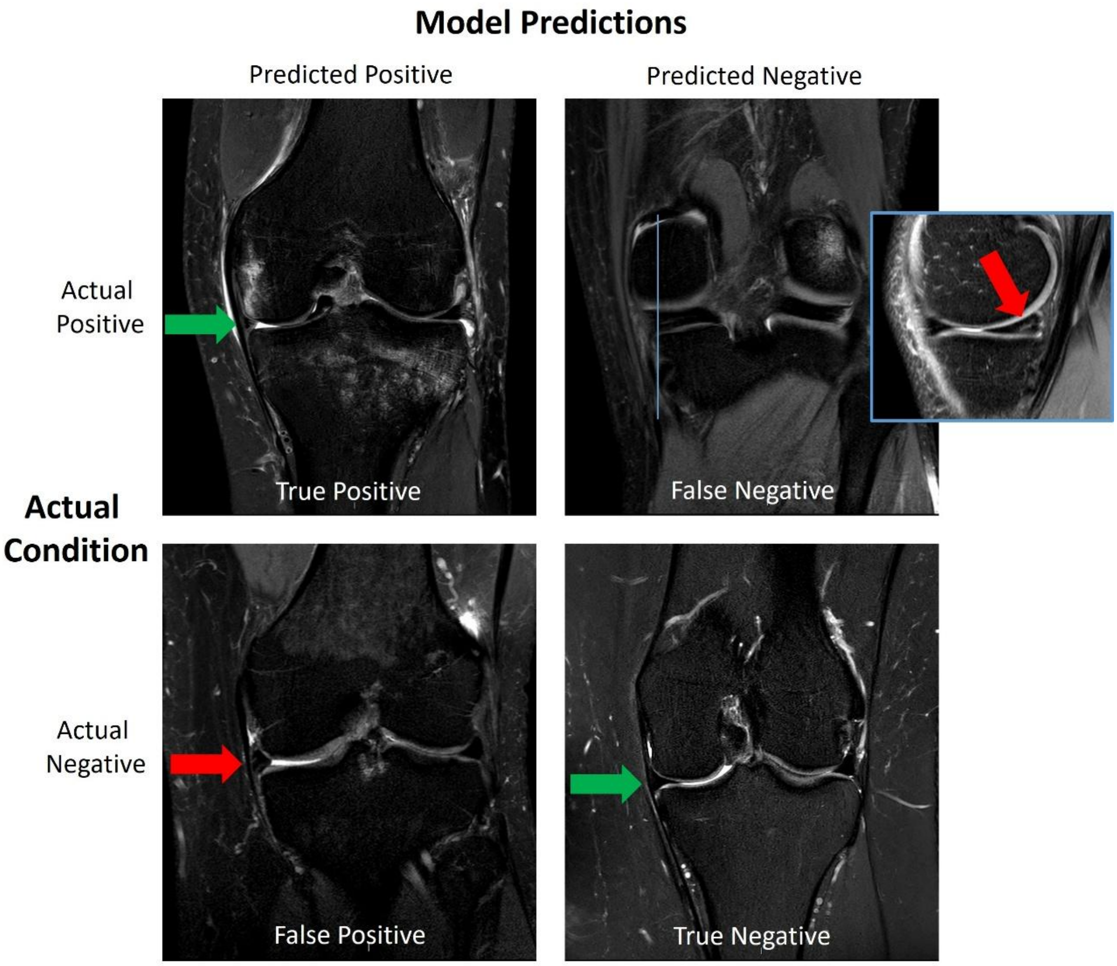

Supplementary Figure 3: Model predictions vs. ground truth for *medial meniscus tear*. Figure organization as in Figure 4.

- True Positive: The model correctly identified the presence of a medial meniscus tear. This was a bucket-handle tear, with the peripheral rim remaining in situ (green arrow) and the remaining meniscus body flipped into the intercondylar notch. Associated femoral and tibial bone bruise and a complete rupture of the anterior cruciate ligament.
- True Negative: The model correctly identified the absence of a medial meniscus tear. Regular wedge shape and hypointense signal intensity of medial meniscus (green arrow).
- False Negative: The model failed to identify a medial meniscus tear when it was present. Oblique tear reaching the meniscal undersurface (red arrow), not visible on coronal slices and only detectable on sagittal slices. The blue vertical line indicates the sagittal slice of the inset box.
- False Positive: The model incorrectly predicted a medial meniscus tear when there was none. While the apex of the meniscus body was truncated (red arrow) and degenerative meniscopathy was present in the posterior horn (not shown), no definitive tear was discernible.
